# Errors in structural biology are not the exception

**DOI:** 10.1107/S2059798322011901

**Published:** 2023-02-27

**Authors:** Yunyun Gao, Volker Thorn, Andrea Thorn

**Affiliations:** a Insitut für Nanostruktur und Festkörperphysik, Universität Hamburg, Luruper Chaussee 149, 22761 Hamburg, Germany; b 90489 Nürnberg, Germany; Institute of Integrative Biology, University of Liverpool, United Kingdom

**Keywords:** errors, structural biology, modelling, quality indicators, validation

## Abstract

During the COVID-19 pandemic, the structural biology community swung into action quickly and efficiently, and many urgent questions were solved by macromolecular structure determination. The Coronavirus Structural Task Force evaluated all structures from SARS-CoV-1 and SARS-CoV-2, but errors in measurement, data processing and modelling are present beyond these structures and throughout the structures deposited in the Protein Data Bank. Identifying them is only the first step; in order to minimize the impact that errors have in structural biology, error culture needs to change. It should be emphasized that the atomic model which is published is an interpretation of the measurement. Furthermore, risks should be minimized by addressing issues early and by investigating the source of a given problem, so that it may be avoided in the future. If we as a community can do this, it will greatly benefit experimental structural biologists as well as downstream users who are using structural models to deduce new biological and medical answers in the future.

## Abstract 2

Errors from measurement, data processing and modelling are present throughout structures deposited in the Protein Data Bank. Identifying them is only the first step. What needs to change in order to minimize their impact?

## 1. Introduction

Atomic structures of biological macromolecules enable us to understand how cells work or to explain mechanisms of disease on the molecular scale, for example in the ongoing COVID-19 pandemic. Macromolecular structures deposited in the wwPDB (Berman *et al.*, 2003 ▸) also serve as a basis for downstream usage, for example as training data for fold prediction in *AlphaFold*2 (Jumper *et al.*, 2021 ▸) or *RoseTTAFold*2 (Baek *et al.*, 2021 ▸), as starting models for molecular dynamics (Karplus & Petsko, 1990 ▸) or for structure-based drug design (Klebe, 2000 ▸). However, these structures, which are obtained by NMR, macromolecular crystallography (MX) or 3D electron cryo-microscopy (cryo-EM), are not direct experimental observations themselves, but are merely interpretations: models that are built to be as consistent as possible with the observed data and with *a priori* knowledge about sequence and chemical geometry. As a consequence, the structures and the information that we can derive from them are only as good as the (limited) understanding of the underlying principles, and are prone to incorrect judgements. These are the ‘errors’ discussed here: mistakes – unintentional, objectively wrong judgements, where we as scientists could have done better. Intentional misconduct (Borrell, 2009 ▸) or the technical limitations of our methods to deduce answers to biological questions (of which there are still many) are not within the scope of the article.

During the COVID-19 pandemic, structural biologists recognized the enormity of the challenge and responded very rapidly to solve the structures of the 28 proteins encoded by the SARS-CoV-2 genome in order to understand the viral life cycle and to enable structure-based drug design. Over 2000 structures of most of the viral proteins and their complexes were released in a span of a few months. These structural models serve as a basis for research to understand how the virus hijacks human cells, for structure-based drug design and to aid in the development of vaccines. However, errors occur in even the most careful structure determination. The Coronavirus Structural Task Force responded to this challenge by rapidly categorizing, evaluating and reviewing all of these structures in order to help downstream users and the original authors (Croll, Diederichs *et al.*, 2021 ▸). They analysed the quality of the atomic models, of the experimental data and their processing both automatically as well as, for selected cases, by hand. Most senior members of the task force are not only expert structure solvers but also methods developers, which gives them a unique advantage: they understand the computational side of structure solution very well and are able to differentiate between user errors, technical limitations and artefacts that result from the methods employed (although the boundaries can be fluid).

**Figure 1:**
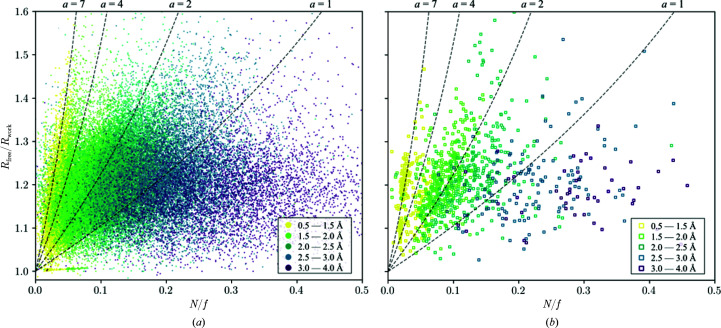
Scatter plots of *R*_free_/*R*_work_ against *N*/*f* for (*a*) all PDB entries with a resolution better than 4.0 Å and (*b*) *Sarbecovirus* structures. *N* is the number of atoms included in the refinement and *f* is the number of reflections used. The four dotted lines with different values of *a* represent the *R*_free_/*R*_work_ ratios which should be achievable at the end of a refinement when only random uncorrelated errors exist and are defined by [(1 + *aN*/*f*)/(1 − *aN*/*f*)]^1/2^ (Tickle *et al.*, 2000 ▸). *a* corresponds to the number of independent parameters being determined per atom, with a lower value of *a* corresponding, for example, to isotropic refinement with highly weighted restraints and a high value of *a* corresponding to anisotropic refinement with lower weighted restraints. The colours encode the corresponding resolution ranges of the PDB entries. The distributions are rather similar and show that there is over-refinement and under-refinement (outliers of a resolution group shifted left or right from the majority of the distribution along the *a* curve, respectively).

Systematic inspection revealed that errors are not the exception, with their impact on direct conclusions and downstream work being very varied (Croll, Diederichs *et al.*, 2021 ▸). At first, it was assumed that these errors were the result of the rapid solution of these structures in the face of the global COVID-19 pandemic, but statistical analyses showed that the model–data discrepancy (as measured by *R* values) in *Sarbecovirus* X-ray structures was on a par with others deposited in the PDB, suggesting an astonishing robustness of modern crystallography pipelines (see Fig. 1 ▸). However, systematic under/over-refinement of deposited models is also consistently observable, especially for medium- to low-resolution structures. Moreover, global cross-validation by *R*_free_ does not guarantee an error-free model. This being said, all structures leave room for improvement.

## 2. Results

### 2.1. Why are errors so common?

**Figure 2:**
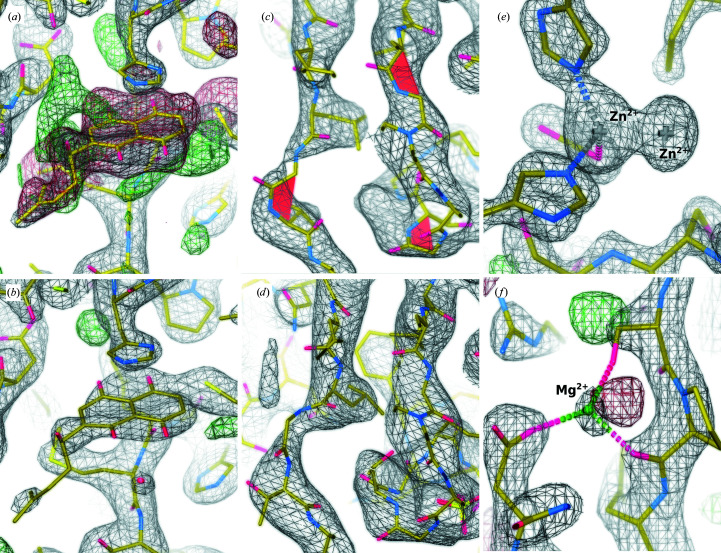
Errors in model building and refinement. (*a*) Shikonin in SARS-CoV-2 main protease (PDB entry 7ca8; Li *et al.*, 2021 ▸). The *B* factors of the ligand are set uniformly to 20 Å^2^, whereas those of the surrounding residues are around 50 Å^2^. Difference map (green and red) contour level 0.248, r.m.s.d. 3.123 Å; 2*mF*_o_ − *DF*_c_ electron-density map (grey) contour level 0.306, r.m.s.d. 1.687 Å. (*b*) The re-refined model in (*a*) with proper ligand *B* factors. Difference map contour level 0.257, r.m.s.d. 3.509 Å, 2*mF*_o_ − *DF*_c_ electron-density map contour level 0.249, r.m.s.d. 3.409 Å. (*c*) Nonproline *cis*/twisted peptide bonds (red) located in SARS-CoV-2 helicase (PDB entry 6jyt; Jia *et al.*, 2019 ▸). Map contour level 0.147, r.m.s.d. 1.104 Å. (*d*) Changing the *cis*-peptide bonds to a more plausible backbone conformation improves the density fit of (*b*). Map contour 0.117, r.m.s.d. 0.609 Å. (*e*) The blob associated with coordinated Zn^2+^ is assigned as a free Zn^2+^ ion, which is chemically not possible, and the deposited structure (PDB entry 6vyo; Center for Structural Genomics of Infectious Diseases, unpublished work) was later updated with a chloride ion next to the coordinated zinc ion instead. Map contour level 1.005, r.m.s.d. 2.711 Å. (*f*) A magnesium ion in SARS-CoV-2 endoRNase (PDB entry 6vww; Kim *et al.*, 2020 ▸). The coordination geometry (tetrahedral) and bond valence (0.5) are indicated as outliers by *CheckMyMetal* (Zheng *et al.*, 2017 ▸); they are expected to be octahedral and 2.0, respectively. A water molecule would be more plausible. Map contour level 0.569, r.m.s.d. 2.620 Å; difference density at contour level 0.108, r.m.s.d. 3.000 Å.

This is not always the fault of structural biologists. It lies in the nature and the complexity of structure determination: the true point of convergence of the fit between molecular models and measured data in crystallography or cryo-EM is unknown to the scientists or any refinement program. To obtain an atomic model, manual intervention is always needed, which requires expertise in many different aspects: sample setup and measurement, data processing to refinement, the chemistry and biochemistry of proteins, and the idiosyncrasies and usage of the software employed. Fig. 2 ▸ shows some examples of common errors in the step of model building and refinement from published *Sarbecovirus* structures.

### 2.2. What are the consequences of errors in macromolecular structures?

Errors in structure solution have both immediate and long-term effects. The worst case for the experimentalist is a complete biological misinterpretation, or an invalid answer to a medicinal question. Such errors may be treated as concerns of misconduct and could lead to the retraction of papers and significant loss of credibility in the community; examples have been reported by the IUCr Editorial Office (2010 ▸) and can be found at https://www.nature.com/collections/prbfkwmwvz/ and https://retractionwatch.com/. However, errors with less immediate significance tend to be retained and propagate as structures are used in molecular replacement or docking. For example, when a structure has been solved for the first time, such as the SARS-CoV RNA polymerase complex (PDB entry 6nur; Kirchdoerfer & Ward, 2019 ▸), the structure is often used as a template for all subsequent structures. PDB entry 6nur was used directly or indirectly as a template for at least 12 other SARS-CoV-2 structures. However, there was a nine-amino-acid out-of-register error at the C-terminus which interacts with the RNA after a loop which had no density in the reconstruction map. The C-terminus of the original structure did not allow the identification of side chains. This error was perpetuated through to all later structures of *Sarbecovirus* RNA polymerases, even when the map resolution became better and side chains could be identified. Details of this case have been published (Croll, Diederichs *et al.*, 2021 ▸; Croll, Williams *et al.*, 2021 ▸), after which the PDB entries were mostly corrected.

We also tend to forget that 99% of users of the Protein Data Bank (PDB) are people who are not depositors themselves (Burley *et al.*, 2018 ▸). Many of these users can be assumed to be structural bioinformaticians, who use models as basis for fold prediction, molecular-dynamics simulations, drug design *etc*. The most prominent example of such an application is as training data for *AlphaFold* (Jumper *et al.*, 2021 ▸). The reliability of AI-based *ab initio* fold predictions such as *AlphaFold* depends directly on the correctness of the training data, *i.e.* the structures deposited in the PDB, and few PDB structures are entirely ‘correct’ (Croll, Diederichs *et al.*, 2021 ▸; Read *et al.*, 2011 ▸). Therefore, it is of crucial importance to avoid certain errors and to improve structures so that we can extract as much biological meaning from the measured data as possible.

### 2.3. Cost analysis of errors

The cost of errors can be material and immaterial, and may include money, man-hours or scientific reputation. It typically multiplies with each step from measurement to biological conclusions as the error remains undetected. Imagine a worst-case scenario, in which a drug binding site is mismodelled (Chakraborti *et al.*, 2021 ▸), for example leading to a different hydrophobicity. At first, the cost would be fairly small if the problem is detected and corrected during model building and routine validation (Chen *et al.*, 2010 ▸; Read *et al.*, 2011 ▸; Sippl, 1993 ▸; Vriend & Sander, 1993 ▸). However, if it is discovered after deposition in the PDB, all tables and statistics would need to be remade as the structure has to be re-refined. If an article discussing the structure containing the error was published, there would be an even larger loss in publication and writing costs, and the reliability of the conclusion would suffer as well. From this point forwards, the authors may find it undesirable to correct the structural model for fear of loss of reputation.

If the error remains undetected at this point, and the structure is used for *in silico* structure-based drug design, it may lead to the development of a drug candidate, which is then synthesized and then, finally, shows no high affinity for the structure in binding studies. At this point, not only are the material and immaterial costs of the mistake vastly increased, it is also more difficult to find the original source of the problem, and the original author. In industry, there is an ‘empirical rule of ten’, a rule of thumb that a tenfold increase in cost occurs at every manufacturing stage in which a product defect is not detected and addressed (Tönnes *et al.*, 2016 ▸; Pfeifer, 2001 ▸).

### 2.4. New error culture

The risk of introducing an error with fatal consequences for the biological conclusion of a paper is relatively low for the individual principal investigator, and hence the personal cost of making errors is mostly low (perhaps with the exception that errors can obstruct a structure solution completely). However, errors in deposited models accumulate damage downstream, both in terms of work time and research money, for example when a mismodelled drug binding site is the subject of structure-based drug-design studies.

It is therefore prudent to recognize and address these problems from the start. In order for this to happen, the structural biology community needs to change its error culture, *i.e.* how we deal with mistakes and their consequences. Unfortunately, personal and individual blame are all too easily applied to those whose errors become public. The authors would like to propose the following directives, which are derived from current practices in production quality control (Masing & Bläsing, 1999 ▸; Nakajima, 1988 ▸).

(i) Those who commit errors should regard errors as solvable problems. Structural models are only one possible interpretation of the data, and therefore can be changed and evolve. Better interpretations should be praised.
(ii) Methods developers and senior scientists should be role models in how they take responsibility for their own errors, deal with them and adapt hypotheses accordingly. It is important to inspire a desire to learn instead of a fear of failure.
(iii) An objective and neutral communication about errors as well as infrastructure to facilitate this communication are necessary. One could say we should ‘blame structures, not people’.
(iv) Error correction should be seen as beneficial not only for scientific integrity but also to advance scientific practice. Most identified errors, even the idiosyncratic ones, have the potential to be utilized to improve the systems that we use and, along with this, reproducibility.

Certainly, there are obstacles to these changes. According to our experience, researchers are often discouraged from seeking advice on challenging structural solutions from experts, for fear of being scooped or being seen as incompetent. Group leaders play a central role in this and may themselves feel that it is inadequate to seek advice before deposition or publication. However, early detection of errors can be highly beneficial, as errors may point to underlying problems which need addressing. Molecular models are often treated as objective ‘absolutes’ in structural biology publications, but rather are interpretations of the experimental data. Raw data, instead of their interpretation, provide the evidence for the scientific result: the credibility of the model is determined by the quality of the data and also by the methods and logic connecting data and interpretation. Scientific progress demands that scientists challenge this logic.

### 2.5. Dealing with errors

If we can change the error culture as described above, errors in our structures will become a valuable resource instead of being a burden. Therefore, in the last section of this paper, we will discuss strategies for dealing with errors in a constructive manner.

As a general rule, fixable errors can be separated into two classes: (i) errors caused by processes and (ii) random errors. Processes in this context means a series of steps taken in order to achieve a particular end, such as the method of cryo-cooling, measurement strategy, scaling algorithm or refinement program employed. Errors that can be fixed by adjusting the process can be dealt with as follows. Firstly, the error has to be identified and its cause has to be found by analysing and evaluating the processes and workflows involved. This can, for example, be performed by the ‘Five Whys’ (Ōno, 1988 ▸), where one asks ‘why’ until the underlying source of a problem has been found. Secondly, the cost of an error has to be determined both in terms of its impact and its frequency. There are errors that are very rare or have no great impact (not even in the downstream usage of a structure). It is only reasonable to combat errors if they have significant impact or occur often, such as a metal mis-assignment in a catalytic centre, a failure to assign the correct chirality to a glycosidic bond or the introduction of two domains on a different scale when docking into a cryo-EM reconstruction map (Croll, Diederichs *et al.*, 2021 ▸; Mostosi *et al.*, 2020 ▸). After the error has been identified, its source determined and its cost established, and it has been decided that it needs to be addressed, measures must be implemented to eliminate the risk of recurrence. In structural biology, this means finding new best practices, changing processes such as sample setup or user interfaces in the software *etc.* Luckily, this problem solving is what we as scientists all excel at, and it is often the most fun part of error management. However, it may be necessary to contact beamline scientists, mailing lists or software developers, which therefore should be strongly encouraged from an early career stage. After a solution to avoid the error in the future has been found, a last and very important step is necessary: it has to be checked that the corrective action which was implemented is actually working, and repetition is actually avoided! Where possible, the effectiveness of these measures should be monitored. It is always useful to record such measures and their justification in writing.

Errors that are not caused by processes are much more difficult to address. They can only be combatted by user education, by combining expertise and by sharing experience. In a laboratory environment this can be stimulated by communication, teamwork and a setting that encourages critical thinking, although it works to our detriment that scientific achievements and research are still considered more as individual achievements than as the result of teamwork. Without the will of the principal investigators, a continuous improvement of processes and thus of results cannot be achieved. The prerequisite for these positive changes is a culture of openness and dialogue, where improvement is emphasized, blame is minimized and all team members are able to communicate and criticize openly.

## 3. Conclusion

The Coronavirus Structural Task Force evaluated structures from SARS-CoV-1 and SARS-CoV-2 using a bespoke pipeline and expert knowledge. It has been demonstrated both by them (Croll, Diederichs *et al.*, 2021 ▸; Nolte *et al.*, 2022 ▸) and by others (Shao *et al.*, 2017 ▸; Lütteke & von der Lieth, 2004 ▸; Joosten *et al.*, 2012 ▸; Read *et al.*, 2011 ▸; Chen *et al.*, 2010 ▸; Wiederstein & Sippl, 2007 ▸; Agirre *et al.*, 2015 ▸; de Vries *et al.*, 2021 ▸) that errors in experimental structure solutions are very common and many of these errors are systematic, *i.e.* caused by processes. Identifying them is only the start of addressing them by adjusting processes. Communication, user education and teamwork are absolutely necessary to minimize the amount of errors. If we can do this, it will greatly benefit us as experimental structural biologists and downstream users who are using structural models to deduce new biological answers. We should therefore strive to address errors together as a community with a view to a healthy, solution-oriented culture. This also means that we need to understand that, while molecular models are central to structural biology publications, they are a mere interpretation, and we should allow these hypotheses to be challenged. This is helped by the PDB in many ways, with experimental data deposition, public and open accessibility, validation reports and recently even the acceptance of revisions of entries. This could be facilitated even more on an infrastructure level by allowing raw data deposition, questions about structure depositions or corrections to be proposed by third parties and the original author being able to answer these questions or ‘pull’ these requests to update their structure in a quick and easy manner. As we as structural biologists improve our ability to model macromolecular structures with a better fit to experimental data, and understand more about errors in our measurements and data processing, errors will become less frequent and completely automatic structure solution will finally become achievable.
